# Identification of JAZ-interacting MYC transcription factors involved in latex drainage in *Hevea brasiliensis*

**DOI:** 10.1038/s41598-018-19206-3

**Published:** 2018-01-17

**Authors:** Jinling Zhai, Hui Hao, Hua Xiao, Yuxin Cao, Xiangui Lin, Xi Huang

**Affiliations:** Hainan Key Laboratory for Sustainable Utilization of Tropical Bioresources, Institute of Tropical Agriculture and Forestry, Hainan University, Haikou, 570228 P. R. China

## Abstract

*Hevea brasiliensis* Müll. Arg. is one of the most frequently wounded plants worldwide. Expelling latex upon mechanical injury is a wound response of rubber trees. However, JA-mediated wound responses in rubber trees are not well documented. In this work, three JAZ-interacting MYC transcription factors of *H. brasiliensis* (termed HbMYC2/3/4) were identified by yeast two-hybrid screening. HbMYC2/3/4 each showed specific interaction profiles with HbJAZs. HbMYC2/3/4 each localized in the nucleus and exhibited strong transcriptional activity. To identify the target genes potentially regulated by HbMYC2/3/4, *cis*-elements interacting with HbMYC2/3/4 were first screened by yeast one-hybrid assays; the results indicated that HbMYC2/3/4 each could bind G-box elements. Additional analysis confirmed that HbMYC2/3/4 bound the *HbPIP2;1* promoter, which contains five G-box *cis*-elements, and regulated the expression of reporter genes in yeast cells and *in planta. HbMYC2/3/4* were induced by exogenous JA treatment but suppressed by ethylene (ET) treatment; in contrast, *HbPIP2;1* was positively regulated by ET but negatively regulated by JA treatment. Given that *HbPIP2;1* is involved in latex drainage, it could be proposed that HbMYC2/3/4 are involved in the regulation of *HbPIP2;1* expression as well as latex drainage, both of which are coordinated by the JA and ET signalling pathways.

## Introduction

Over 2,000 plant species produce rubber (*cis*-1–4-polyisoprene). Because of its high rubber productivity and rubber quality, *Hevea brasiliensis* Müll. Arg. is the sole commercial source of natural rubber. Rubber is produced and accumulates in latex in the laticifer network of *H. brasiliensis*. The laticifers consist of contiguous anastomosis cells forming a network structure arranged in rings parallel to the vascular cambium, which allow the drainage of latex from a large area of bark by a single tapping^1^. As the cytoplasm of laticifers, latex is harvested by farmers by regularly cutting bark at intervals of 2–3 days. As such, rubber trees become one of the most frequently wounded plants worldwide. Previous reports have shown that mechanical wounding induces laticifer differentiation and latex production. Three decades ago, 2–3-fold more laticifer rings were observed by light microscopy in the exploited trees than in the unexploited trees^2,3^. Additionally, secondary laticifer differentiation can be induced in the stem of epicormic shoots by treatment with exogenous jasmonic acid (JA) or its derivatives^1^. The induction of laticifers could serve as an excellent indicator of the wounding response and latex biosynthesis regulated by JA in rubber trees. Recent reports have shown that the differentiation of secondary laticifers was prevented when the wounding site of epicormic shoots was wrapped immediately after wounding. Wounding-induced laticifer differentiation has been proposed to be correlated with JA accumulation, reactive oxygen species, as well as dehydration at the wounding site^4^. Our recent report also confirmed that local tissue dehydration was a key signal for laticifer differentiation. Dehydration-related genes, such as *HbDHN*s and *HbNAC1*, are differentially expressed on wrapped and exposed wounding sites. Furthermore, HbNAC1 was shown to bind to the cis-element CACG in the promoter region of the gene encoding the small rubber particle protein (SRPP)^5^. Arabidopsis overexpressing *HbDHN*s show higher activity of antioxidant enzymes and accumulate fewer reactive oxygen species (ROS)^6^. Given that ROS have been proposed to represent a key signal for laticifer differentiation^4^, HbDHNs might act as ROS scavengers, directly or indirectly affecting laticifer differentiation. However, how JA signalling is involved in laticifer differentiation and latex biosynthesis is less known.

In Arabidopsis, JA regulates many developmental and metabolic processes, such as vegetable growth, stamen development, senescence, trichome patterning, and anthocyanin biosynthesis^7–9^. JA is also involved in the response to a number of biotic/abiotic stresses, such as necrotrophic pathogens, herbivores, mechanical wounding, UV radiation, ozone, and salinity^10,11^. The JA signalling pathway has been well elucidated in Arabidopsis. Dissection of JA signalling was predominantly dependent on the identification of mutants that are deficient in JA synthesis or perception via a forward genetics approach, among which *coi1* (*coronatine insensitive 1*) is the most important gene. COI1 encodes an F-box protein that associated with other proteins, including SKP1 and CULLIN, to form the SCF^COI1^ ubiquitin–ligase complex^12,13^. The SCF^COI1^ complex binds to target proteins, which are then polyubiquitinated and subsequently degraded by the 26 S proteasome. Another major advance in study of the molecular mechanism of JA signalling was made possible by the identification of the first SCF^COI1^ targets, which compose the jasmonate ZIM-domain (JAZ) protein family^14,15^. JAZ proteins function as repressors of JA signalling to interact with JA-responsive transcription factors (e.g., MYC2) and inhibit their transcription^14,16^. After the perception of wounding signals or developmental cues, JA accumulates and is conjugated with isoleucine, which serves as active form to mediate the interaction between COI1 and JAZ repressors, leading to the ubiquitination of JAZ proteins. The degradation of JAZs results in the release of downstream transcription factors, activating the JA response. The COI1-JAZ-MYC2 complex was proposed to represent the core signalling module in the JA pathway^17^. As the SCF^COI1^ complex is highly conserved in plants^13,17,18^, the spatially and temporally specific expression and alternative splicing as well as the differing repression of target transcription factors of individual JAZ gene members may account for the specific responses of plants to JA signals^17,19^.

MYC2 was the first reported TF regulated by JAZ proteins^14,20^. MYC2 and its closest homologues (MYC3 and MYC4) interact the most with JAZ proteins^21–23^. All three MYC proteins belong to group IIIe of the bHLH family; in the members of this family, five different domains have been identified, including a JAZ-interacting domain (JID) at the N-terminus and a conserved ACT-like domain at the C-terminus, in addition to a DNA-binding bHLH domain^24^. Although all three bHLH proteins have similar protein structures, they seem to regulate specific subsets of JA responses. For example, MYC2 is a positive regulator of the JA-mediated inhibition of primary root growth, anthocyanin biosynthesis, and oxidative stress tolerance but is a negative regulator of JA-mediated resistance to necrotrophic fungi^20,25^; on the other hand, MYC3 and MYC4 are important for JA-mediated resistance to the herbivore *Spodoptera littoralis*^24^. The JID domain is also found in several other bHLH proteins, such as GL3, EGL3, and TRANSPARENT TESTA8 (TT8; At4g09820), all of which belong to group III of the bHLH family^26^. GL3, EGL3, and TT8 interconnect with both WD40 proteins and R2R3 MYB proteins to form protein complexes; these complexes then regulate multiple processes, such as the biosynthesis of anthocyanins and proanthocyanidins, the development of trichomes and root hairs, and so on^8,27–29^. Interestingly, the MYC2/3/4 complex is also involved in the JA-mediated induction of anthocyanin biosynthesis^20,23,25^. How and whether MYC2/3/4 interact with GL3/EGL3/TT8 to regulate anthocyanin biosynthesis remains to be resolved.

In *Hevea brasiliensis*, members of the JAZ gene family have been globally cloned and preliminarily characterized^30,31^. The next key step to reveal the JA signalling pathway in *H. brasiliensis* is the identification of transcription factors regulated by JAZ proteins. In this work, a bait vector of HbJAZ1 was constructed, and proteins that interact with HbJAZ1 were screened using yeast two-hybrid assays. Three bHLH proteins (termed HbMYC2, HbMYC3 and HbMYC4) were identified. Further investigation revealed that these HbMYCs interact with promoters containing G-box cis-elements, e.g., HbPIP2;1, which codes for an aquaporin involved in latex drainage^32^. The data provided in this study might fill the gaps of the JA-mediated mechanism of latex biosynthesis and drainage in rubber trees.

## Results

### Identification of the HbJAZ1-interacting transcription factors of H. brasiliensis

**Figure 1:**
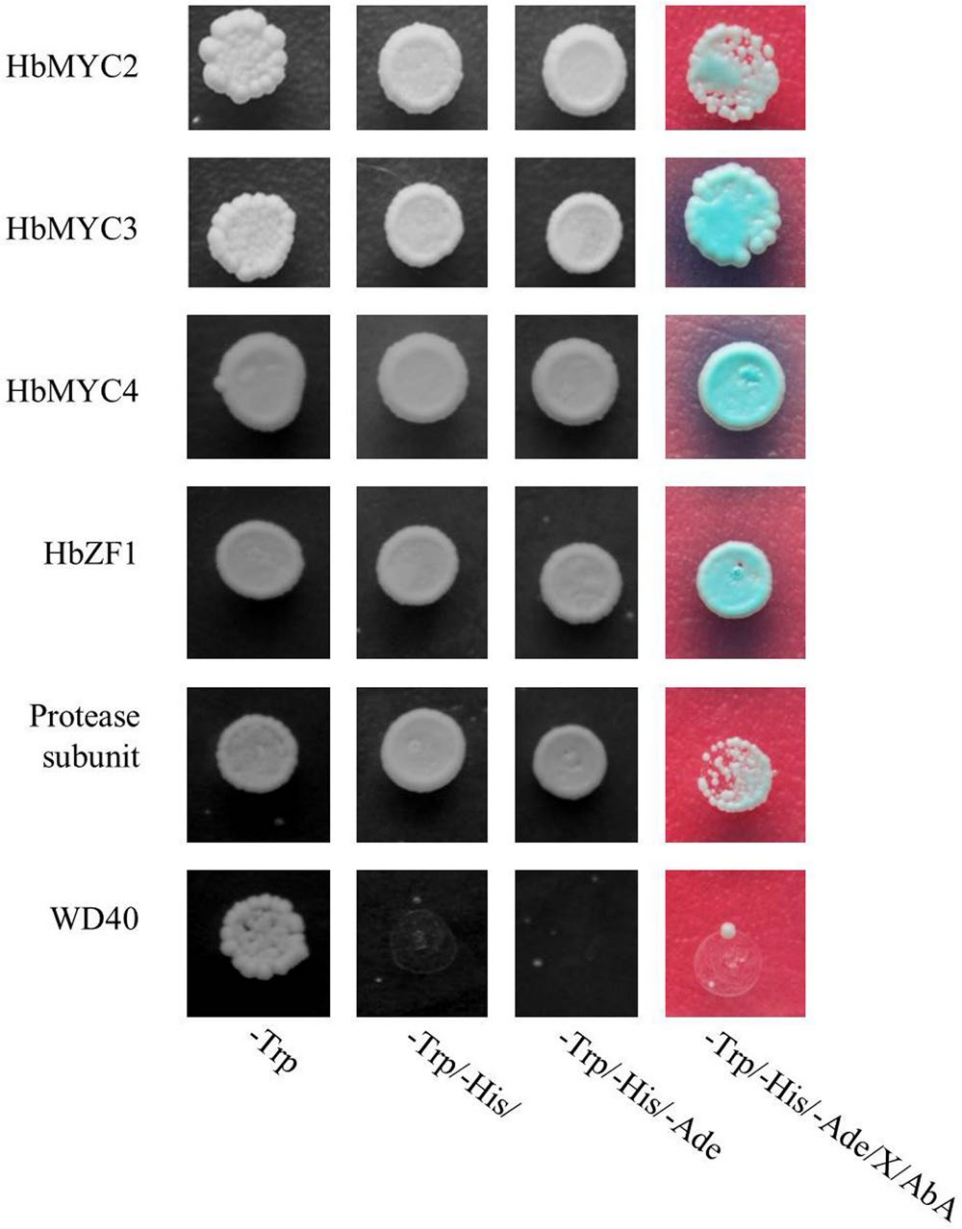
Autoactivation and toxicity test of HbJAZ-interacting proteins. The full-length *Hevea* *HbMYC2*, *HbMYC3*, *zinc-finger*, *protease subunit*, and *WD40* genes were cloned into a pGBKT7 vector to generate bait plasmids, which were subsequently transformed into Y2HGold strains and plated onto plates containing the following media: SD/-Trp, SD/-Trp/-His/, SD/-Trp/-His/-Ade, or SD/-Trp/-His/-Ade/X-α-gal/AbA (125 ng/ml).

To screen the JAZ-interacting transcription factors in *H. brasiliensis*, the full-length ORFs of *HbJAZ1* were inserted into pGBKT7 and transformed into a Y2HGold strain to generate a bait reporter strain. After co-cultivation of the Y2HGold bait reporter strain and the Y187 strain of the Mate & Plate library of *H. brasiliensis*, the cell suspension was plated on QDO/A/X media (quadruple drop-out media: SD/–Ade/–His/–Leu/–Trp + AbA + X-alpha-Gal) to screen for positive colonies. From those colonies, four prey proteins were identified as putative transcription factors, including three basic helix-loop-helix (bHLH) transcription factors and one zinc-finger protein. Two other proteins were annotated as protease subunits and WD40 proteins, which seem to be components of the 26 S proteasome complex and GL3, EGL3, and TT8 protein complexes, respectively. Using *in silico* cloning procedures^31^, the full-length cDNA sequences of these genes were identified, and four TF genes were termed *HbMYC2, HbMYC3, HbMYC4*, and *HbZF1*. The full-length CDSs of the genes were fused into pGBKT7 vectors, which were then transformed into Y2HGold yeast strains. The assays of transcriptional activity showed that the protease subunit and WD40 proteins did not exhibit transcriptional activity but that HbMYC2, HbMYC3, HbMYC4, and HbZF1 exhibited strong transcriptional activity, suggesting that they are putative transcription factors (Fig. 1).

### Phylogenetic analysis of HbMYC2/3/4

**Figure 2:**
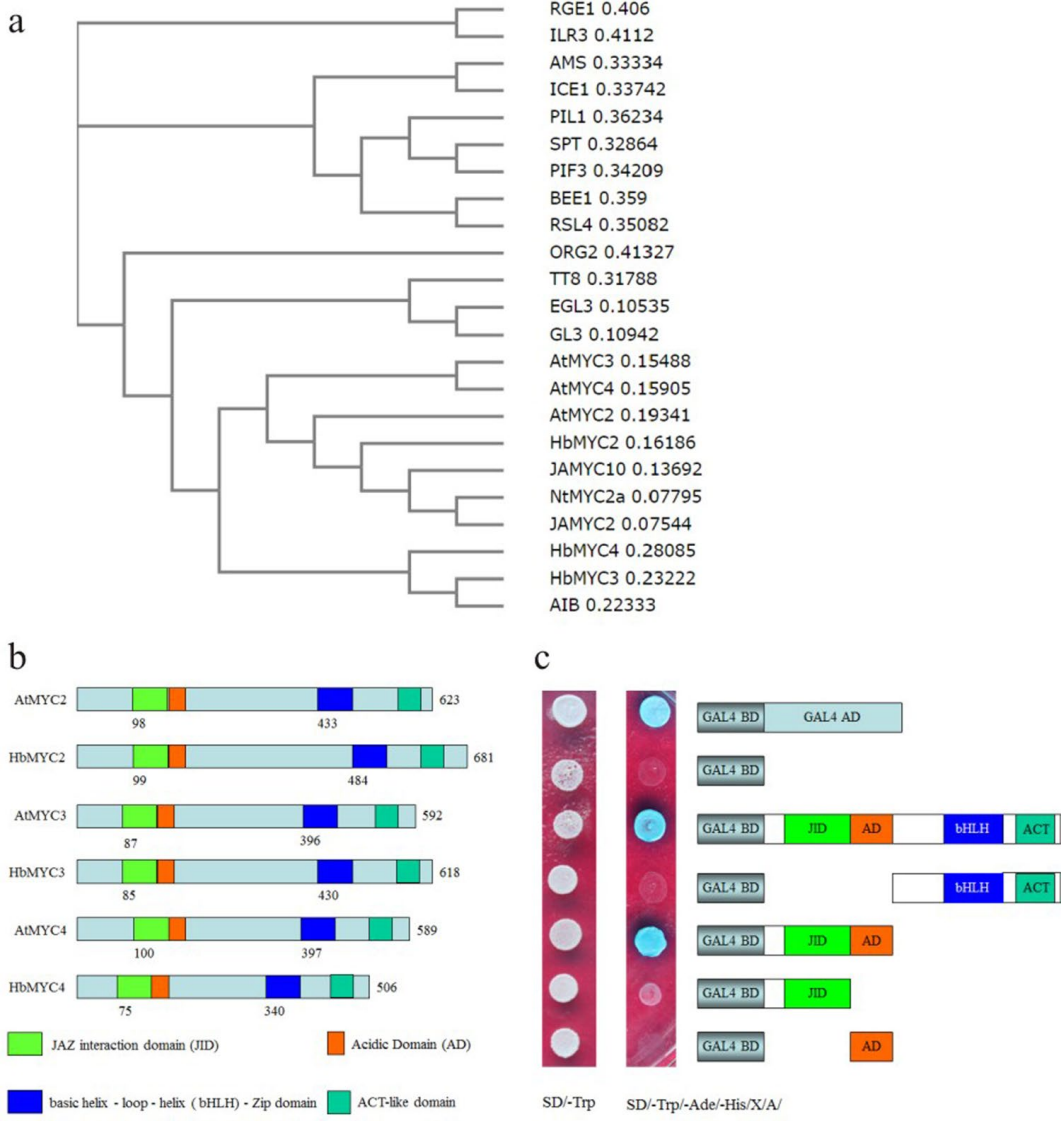
Comparison of the MYC genes of *Hevea brasiliensis* and other bHLH-type transcription factors. (**a**) Phylogenetic tree of the deduced amino acid sequences of HbMYCs and other plant bHLH proteins. The phylogenetic tree was generated based on the alignment of the full-length deduced amino acid sequences of 23 bHLH proteins. Alignment was performed and the phylogenetic tree was constructed by Clustal Omega (http://www.ebi.ac.uk/Tools/msa/clustalo/), with the default settings. (**b**) Domain comparisons between HbMYCs and AtMYCs, JAZ-interacting domains (JIDs), acidic domains (ADs), basic helix-loop-helix (bHLH)-zip domains and ACT-like domains were included. **C**. Transcriptional activity analysis of the different domains of HbMYC3 proteins. Full-length or attenuated fragments of HbMYC3 were inserted into a pGBKT7 vector, after which the vectors were transferred into Y2HGold yeast strains and then plated on SD/-Trp/-Ade/-His/X-α-gal/AbA media.

To classify the HbJAZ proteins that interact with HbJAZ1 in the yeast two-hybrid system, 23 different bHLH proteins were collected for phylogenetic analysis using Clustal Omega online software (http://www.ebi.ac.uk/Tools/msa/clustalo/). These bHLH proteins include JA-responsive MYC2/3/4, TT8, GL3 (GLABRA3) and EGL3 (ENHANCER OF GLABRA3) from Arabidopsis^8,33^; JAMYC2/10 from *Solanum lycopersicum*^34^; NtMYCa from tobacco^35^; ALC (Alcatraz, AtbHLH73), which is required for gynoecium and fruit development^36^; AMS (Aborted Microspores, AtbHLH21), which regulates pollen wall formation^37–39^; ABA-Inducible bHLH (AIB)^40,41^; SPT (Spatula, AtbHLH24), which controls the development of carpel margin tissues^42,43^; ILR3 (IAA-Leucine Resistant 3, AtbHLH105), which modulates iron homeostasis^44^; ICE1 (Inducer of CBF Expression 1, AtbHLH116), which is induced by cold^45,46^; ORG2 (OBP3-Responsive Gene 2, AtbHLH38), which is inducible by salicylic acid^47^; Phytochrome Interacting Factor 3 (PIF3, AtbHLH08) and Phytochrome Interacting Factor 3-Like 1 (PIL1, AtbHLH124)^48–50^; BEE1 (Brassinosteroid Enhanced Expression 1, AtbHLH44)^51,52^; RSL4 (Root Hair Defective 6-Like 4, AtbHLH54)^53^; RGE1 (Retarded Growth of Embryo 1, AtbHLH95); and FIT (Fe-Deficiency Induced Factor 1, AtbHLH29)^54^. Phylogenetic analysis showed that all JA-responsive bHLH proteins, e.g., MYC2, MYC3, MYC4, TT8, GL3, EGL3, JAMYC2, JAMYC10, and NtMYCa, clustered with HbMYC2/3/4, suggesting that these three HbMYCs might also be involved in the JA response. Other bHLH members such as AMS, SPT, ICE1, PIF3, PIL1, BEE1, RSL4, and FIT were classified together, whereas RGE1, ILR3 and ORG2 showed significantly wider genetic distance (Fig. 2a). When further comparing the protein structures, a similar distribution of the domains among MYC2/3/4 and HbMYC2/3/4 was observed. All these proteins contain a JAZ-interacting domain (JID) and an acidic domain (AD) at their N-terminus as well as a bHLH-zip domain and an ACT-like domain at their C-terminus (Fig. 2b). To identify which domain contributed to transcriptional activity, full-length and attenuated fragments of HbMYC3 were inserted into pGBKT7 vectors. These vectors were then transferred into Y2HGold yeast strains, which were subsequently plated onto SD/-Trp/-Ade/-His/X/A media. The full-length and N-terminal fragments containing the JID and AD domains exhibited strong transcriptional activity, which was indicated by strong growth in the SD/-Trp/-Ade/-His/X/A media. On the other hand, the C-terminal fragments containing the bHLH-zip domain and ACT-like domain and those containing only the JID or AD domain did not exhibit transcriptional activity, suggesting that both the JID and AD domains are essential for transcriptional activity (Fig. 2c).

### Interaction between members of the HbMYCs and HbJAZs gene families

**Figure 3:**
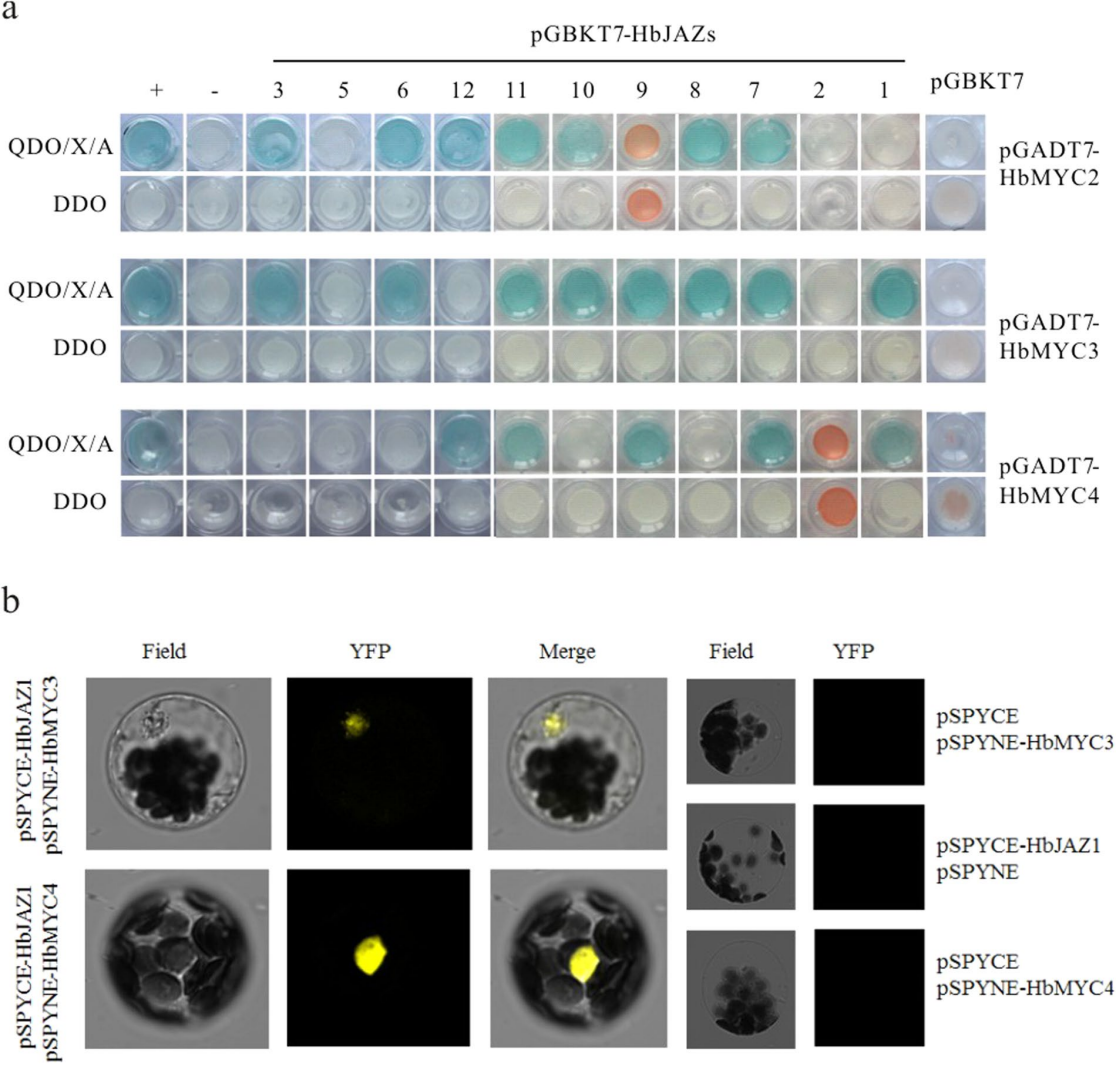
Interaction of HbJAZs with HbMYC proteins. (**a**) Full-length *HbMYCs* and *HbJAZs* were fused into pGADT7 and pGBKT7 vectors to generate prey and bait vectors, respectively, which were further transformed into Y187 and Y2Hgold yeast strains, respectively. The Y187 and Y2Hgold yeast strains were then combined and cultivated. The mating cells were screened on synthetic drop-out (DO) plates (SD-Trp/-Leu), and the Y2H interactions were assessed on QDO/X/A plates (SD-Trp/-Leu/-Ade/-His/X/A). Positive control mating (+): Y2HGold [pGBKT7-53] and Y187 [pGADT7-T]; negative control mating (−): Y2HGold [pGBKT7-Lam] and Y187 [pGADT7-T]. The experiments were performed in triplicate. (**b**) Detection of the interaction between HbJAZ1 and HbMYCs by bimolecular fluorescence complementation. The plasmid combinations of pSPYCE-HbJAZ1 and pSPYNE-HbMYC3 or pSPYCE-HbJAZ1 and pSPYNE-HbMYC4 were co-transferred to *Arabidopsis thaliana* protoplasts, and the yellow fluorescence was observed by laser confocal microscopy.

Given that Arabidopsis MYC2/3/4 interact with different JAZ proteins, the interaction between HbMYCs and HbJAZs was investigated. Full-length *HbMYCs* and *HbJAZs* were fused to pGADT7 and pGBKT7 vectors to generate prey and bait vectors, which were further transformed into Y187 and Y2Hgold yeast strains, respectively. After co-cultivation of the Y187 and Y2Hgold strains, the mated cells were plated onto synthetic drop-out (DO) plates (SD-Trp/-Leu) and QDO/X/A plates (SD-Trp/-Leu/-Ade/-His/X/A). The yeast two-hybrid results showed that HbMYC2 interacted with HbJAZ3/6/7/8/10/11/12 and that HbMYC3 interacted with HbJAZ1/3/6/7/8/9/10/11, whereas HbMYC4 interacted with HbJAZ1/7/9/11/12. The HbMYCs each exhibited individual specific profiles of interaction with HbJAZs, although no correlation of sequence properties was observed. Interestingly, HbMYC2 was screened by the bait protein of HbJAZ1, but the full-length HbMYC2 did not interact with HbJAZ1, suggesting that some sequences of full-length HbMYC2 might inhibit the interaction between HbJAZ1 and the JID domain of HbMYC2, as HbJAZ1 interacted with the attenuated fragment of HbMYC2 in the Mate & Plate library screening (Fig. 3a).

**Figure 4:**
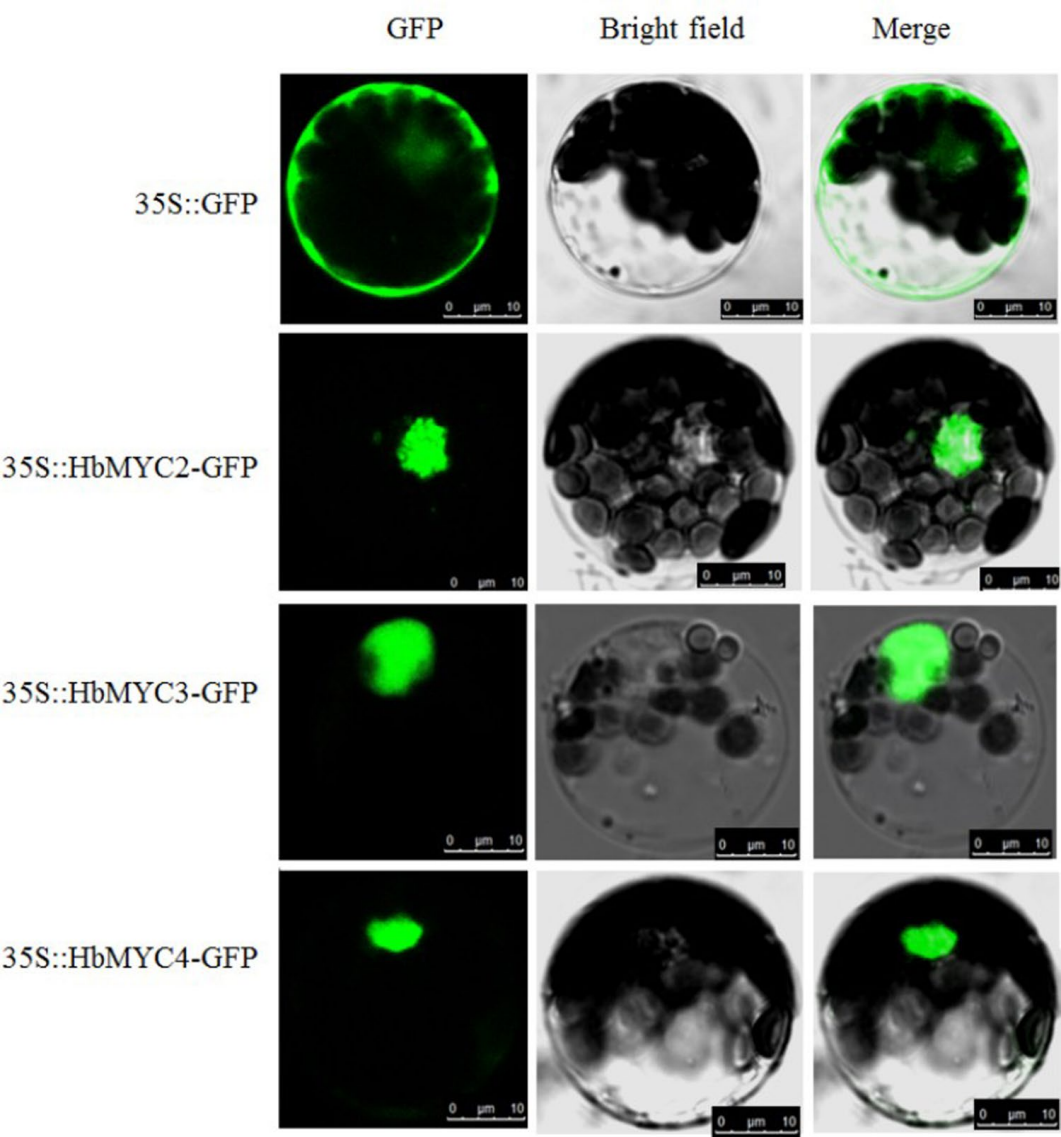
Subcellular localization of HbMYCs. Protoplasts of Arabidopsis leaf epidermis transiently expressing *35 S:HbMYC2-GFP*, *35 S:HbMYC3-GFP* and *35 S:HbMYC4-GFP* fusion proteins observed under a laser scanning confocal microscope.

To verify the confidence of the interaction between HbJAZs and HbMYCs, bimolecular fluorescence complementation (BiFC) assays were performed in *Arabidopsis* mesophyll cells. The combinations of pSPYCE-HbJAZ1 and pSPYNE-HbMYC3 or the combinations of pSPYCE-HbJAZ1 and pSPYNE-HbMYC4 were co-transferred to protoplasts of *Arabidopsis thaliana* and subsequently observed by laser confocal microscopy. No YFP fluorescence was observed when only one of the two proteins was fused to an unfolded YFP fragment; however, when pSPYNE-HbMYC3 or pSPYCE-HbJAZ1 was co-transformed with pSPYCE-HbJAZ1 into Arabidopsis protoplasts, a strong YFP signal was detected (Fig. 3b). Additionally, a YFP signal was significantly localized in the nucleus, suggesting that HbJAZ1 interacts with HbMYCs in the nucleus. The subcellular localization results also confirmed that all three HbMYCs localized in the nucleus (Fig. 4). Considering that HbMYC proteins exhibited strong transcriptional activity (Figs 1 and 2) and localized in the nucleus (Figs 3 and 4), these proteins could be regarded as transcription factors.

### HbMYC2/3/4 proteins specifically bind to the G-box

**Figure 5:**
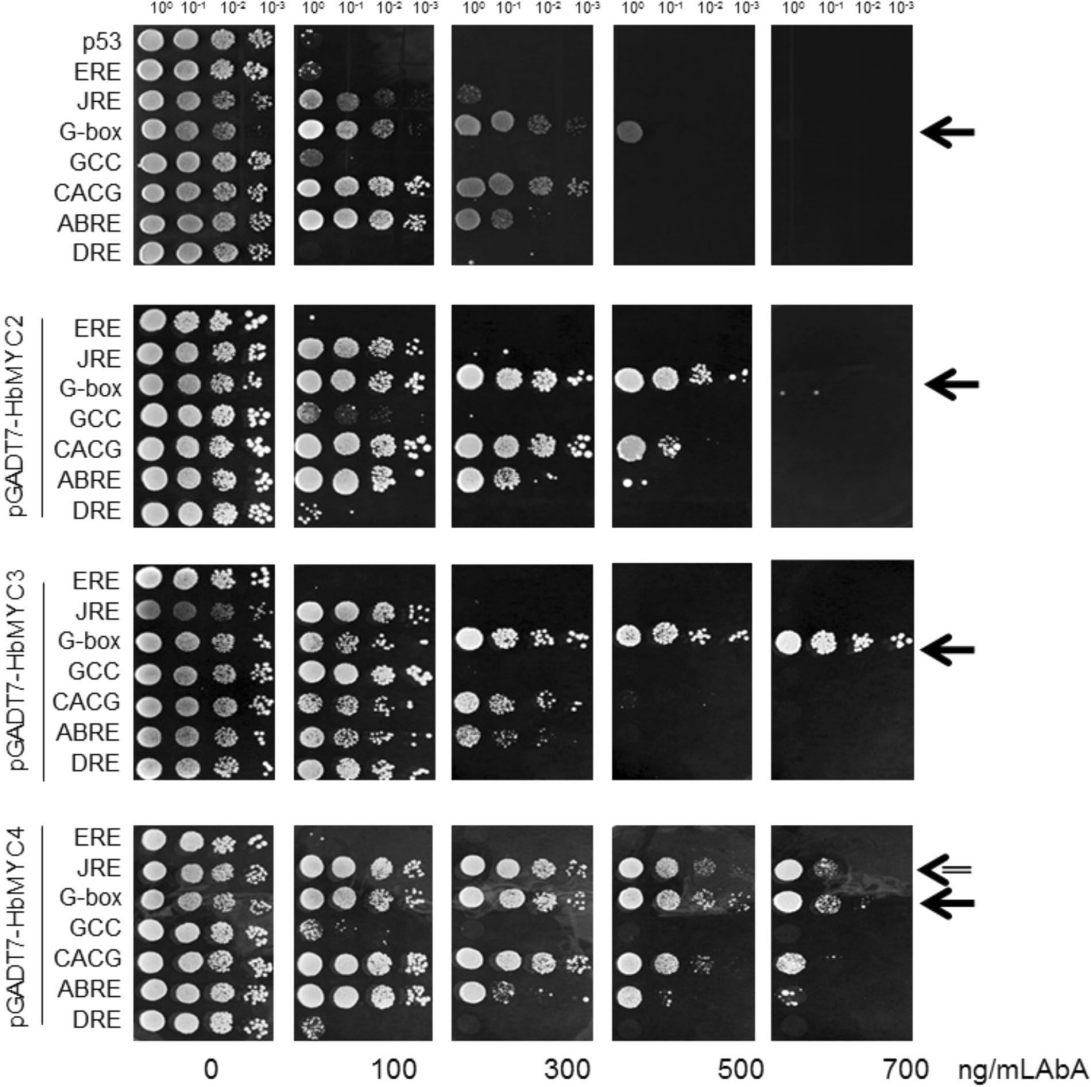
Interaction between HbMYCs and cis-elements by yeast one-hybrid assays. pGADT7-Rec2-*HbMYC2*, pGADT7-Rec2-*HbMYC3* and pGADT7-Rec2-*HbMYC4* were transferred into Y1HGold strains containing different pAbAi-cis-element bait vectors. The strains were then plated on SD/-Leu/AbA solid media containing 0–700 ng/mL AbA to cultivate for four days at 30 °C, after which the growth of the cells was observed. Arrows indicate significant differences in the pAbAi-G-box vector.

To identify the target genes controlled by HbMYC2/3/4, *cis*-elements such as JREs, GCC boxes, ABREs, EREs, G-boxes, CACG boxes, and DREs were inserted into the MCSs of pAbAi vectors. The bait vectors were subsequently co-transformed with pGADT7-HbMYC2, pGADT7-HbMYC3, and pGADT7-HbMYC4. The yeast one-hybrid assay results showed that co-transformation of the pAbAi-G-box bait strain with pGADT7-HbMYC2, pGADT7-HbMYC3, and pGADT7-HbMYC4 significantly enhanced aureobasidin A (AbA) concentrations in the resistant cells, suggesting that HbMYC2/3/4 can bind G-box elements and activate the expression of the *AUR1-C* gene, an antibiotic resistance gene that provides resistance to AbA (Fig. 5). Co-transformation of HbMYCs into bait strains containing GCC boxes, ERE boxes, ABREs, and DREs did not significantly enhance AbA resistance levels, suggesting that HbMYC2/3/4 did not bind these cis-elements. Interestingly, HbMYC4 but not HbMYC2 and HbMYC3 bound JRE and evidently increased the AbA resistance level (Fig. 5).

### HbMYC2/3/4 proteins bind to the promoter and regulate the expression of the HbPIP2;1 gene

**Figure 6:**
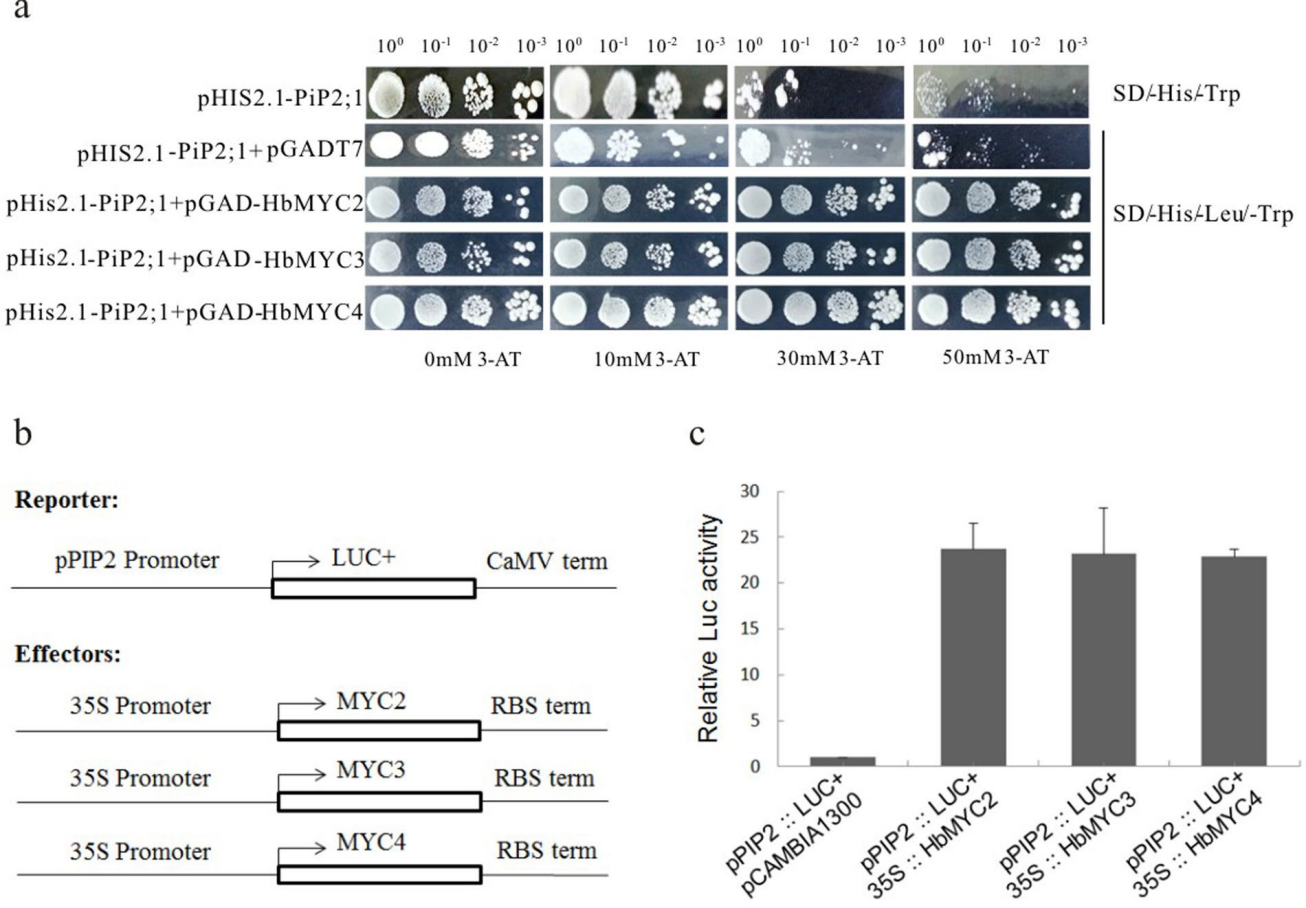
HbMYC2/3/4 bind the promoter and regulate the expression of the *HbPIP2;1-P* gene. (**a**). The pGADT7-*HbMYC2*, pGADT7-*HbMYC3*, pGADT7-*HbMYC4* plasmids were transformed into Y187 Gold strains containing pHis2.1-PIP-P, after which the strains were cultured in SD/-His/-Leu/-Trp + 3-AT media at 30 °C for 3–5 days. (**b**) Schematic diagram of the reporter and effector constructs used in the luciferase assay. The firefly luciferase (LUC) reporter was driven by the HbPIP2;1 promoter, and *HbMYC2/3/4* were driven by the CaMV 35 S promoter in each of the effector constructs. **c**. Luciferase assay of the enhancement of the *HbPIP2;1* promoter activity by the overexpression of HbMYC2, HbMYC3, and HbMYC4 in protoplasts. The pPIP2:LUC reporter and respective 35 S:*MYC2/3/4* effector constructs as well as empty vector controls were co-transformed into *Arabidopsis* protoplasts. Luciferase activities were quantified using a dual-luciferase assay kit (Promega, USA) and detected by using a GloMax® 96 microplate luminometer (Promega, USA). The values are the means ± SDs from the results of three replicates.

To identify the targets genes of HbMYCs, we first screened which latex biosynthesis- or drainage-related gene promoters contained a G-box, which led to the identification of HbPIP2;1 as a potential target of HbMYCs. HbPIP2;1 has been proposed to be one of two aquaporins involved in ethylene stimulation during latex production by regulating the water exchange between inner liber and latex cells in *Hevea brasiliensis*^32,55^. The promoter of* HbPIP2;1* contains 5 G-box core sequences, e.g., CACGTG, CAGACGTGGCA, TACGTG, CACGTC and CACATGG, which are distributed at 128 bp, 240 bp, 434 bp, 373 bp and 64 bp upstream of the ATG translation start site, respectively. The 970 bp promoter sequence upstream of the ATG translation start site of HbPIP2;1 was inserted into the MCS of the bait vector pHis2.1. Co-transformation of HbMYC2/3/4 significantly increased cell tolerance to 3-amino-1,2,4-triazole (3-AT), suggesting that HbMYC2/3/4 bound the *HbPIP2;1* promoter and activated the expression of the *His3* reporter gene (Fig. 6a). To further investigate whether HbMYC2/3/4 could bind the *HbPIP2;1* promoter and regulate gene expression *in planta*, the *HbPIP2;1* promoter was inserted to the MCS of the pSP-luc+NF plasmid (accession U47123). Additionally, the full-length CDSs of HbMYC2/3/4 were inserted into the MCSs of pCAMBIA1300 vectors under the control of the 35 S promoter. Two types of plasmids were co-transformed into protoplasts via PEG-mediated methods. Luciferase activity under the control of the *HbPIP2;1* promoter was significantly elevated after co-transformation with 35 S::*HbMYC2*, 35 S::*HbMYC3*, or 35 S::*HbMYC4*, suggesting that HbMYC2/3/4 could bind the HbPIP2;1 promoter and up-regulate the expression of the reporter gene in plant cells (Fig. 6b).

### Expression profiles of *HbMYC2/3/4* and *HbPIP2;1*

**Figure 7:**
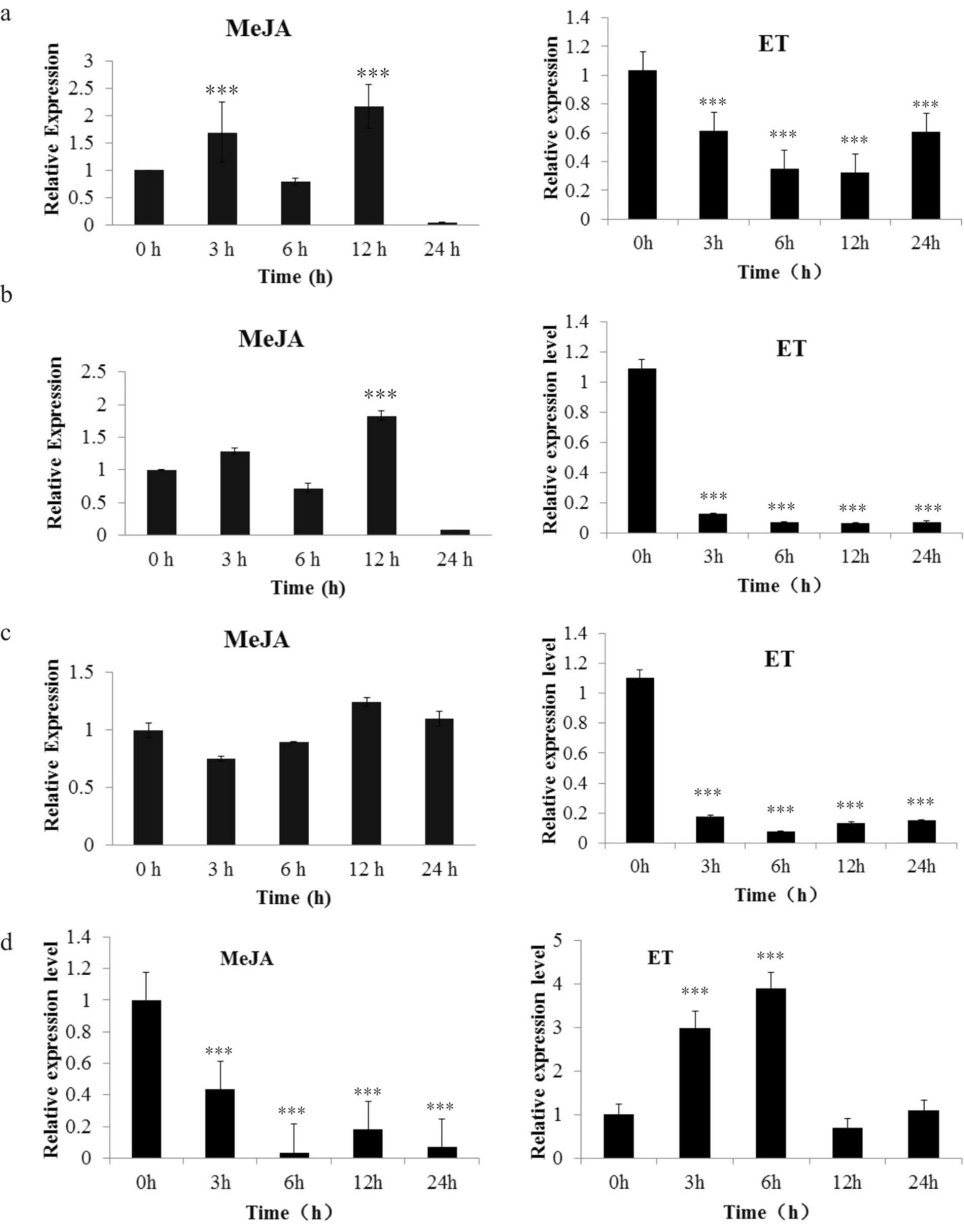
Different expression levels of *HbMYC2/3/4* and *HbPIP2;1* in the MeJA- and ET-treated leaves of rubber trees. Leaves of the epicormic shoots of *Hevea brasiliensis* were sprayed with 100 µM methyl-JA or 100 µM ethrel. Samples were collected at 0 h, 3 h, 6 h, 14 h and 24 h after treatment. Expression levels of the *HbMYC2* (**a**), *HbMYC3* (**b**), *HbMYC4* (**c**) and *HbPIP2;1* (**d**) genes were analysed by qRT-PCR. The values are the means ± SDs from the results of three replicates. Asterisks indicate significant differences compared with those of the 0 h control (***P < 0.005, Student’s t-test).

To investigate how JA and ET signals affect the expression of *HbMYC2/3/4* and *HbPIP2;1*, the leaves of the epicormic shoots of *Hevea brasiliensis* were sprayed with 100 µM methyl-JA or ethrel. The qRT-PCR results revealed that the expression levels of *HbMYC2/3/4* were quickly induced by JA treatment and sharply suppressed by ET treatment, indicating opposite roles for JA and ET in the regulation of *HbMYC2/3/4* (Fig. 7a–c). Surprisingly, the expression profile of *HbPIP2;1* was not consistent with that of *HbMYC2/3/4*. In addition, *HbPIP2;1* was negatively regulated by JA treatment but positively regulated by ET treatment (Fig. 7d), suggesting that there might be other factors coordinating with *HbMYC2/3/4* to regulate the expression of *HbPIP2;1*.

## Discussion

*H. brasiliensis* is a very important crop for natural rubber production. Farmers regularly harvest latex by tapping the bark at intervals of 2–3 days. The drainage and *de novo* biosynthesis of latex is actually a wound response of rubber trees. JA is a master phytohormone that mediates wound responses; these responses have been well elucidated in Arabidopsis and in many other plant species^56^. Discovery of the JAZ gene family has significantly advanced our understanding of how the JA signalling pathway operates and has reinforced the recurring theme that hormone-dependent removal of transcriptional repressors is required for the activation of various plant hormone signalling pathways^57^. More detailed mechanistic understanding came from the identification of JAZ targets using *in vitro* (e.g., yeast two-hybrid) and *in planta* assays^7,8,21^. However, JA-related wound responses have not been well documented in *H. brasiliensis*, and the underlying molecular mechanism is not well known. JA and mechanical wounding have been reported to induce laticifer differentiation^1^. In a previous study, we globally cloned the JAZ gene family of *Hevea brasiliensis*. In the present study, several JAZ targets, including three bHLH proteins (termed HbMYC2/3/4), were identified via yeast two-hybrid screening. All HbMYCs localized in the nucleus and exhibited transcriptional activity, suggesting that they are transcription factors (Fig. 1). Like MYC2/3/4 of Arabidopsis, HbMYC2/3/4 interacted with many HbJAZ members and exhibited specific JAZ interactions profiles (Fig. 3), indicating their specific roles in JA responses. Although two MYC transcription factors have been previously reported to be responsive to multiple treatments in *Hevea brasiliensis*, no evidence has shown whether they interact with JAZ proteins or are related to JA signalling^58^. The identification of HbMYC2/3/4 in this work provides insight into the network of JA signalling in *H. brasiliensis*.

Interaction between *trans*-factors and *cis*-elements is the cornerstone of gene expression regulation. To identify the targets of HbMYCs, the *cis*-elements that could be bound by the HbMYCs were first screened. Seven *cis*-elements, e.g., JREs, GCC boxes, ABREs, EREs, G-boxes, CACG boxes, and DREs, were tested for potential interaction with HbMYCs by yeast one-hybrid assays, leading to the finding that all HbMYCs could bind G-box elements (Fig. 5). In tobacco, NtMYC2 can bind the G-box elements of the promoters of the nicotine biosynthesis-related genes *PMT2* (Putrescine N-Methyltransferase 2) and *QPT2* (Quinolinate Phosphoribosyl Transferase 2), up-regulating their expression^59,60^. Many JA-responsive gene promoters, such as those of *PIN2* and *VSPB* in potato^61,62^, *VSP1* in Arabidopsis^63^, *PMT1a* (Putrescine N-Methyltransferase 1a) in tobacco^64^, *LA* (Leucine Aminopeptidase) of tomato^34^, and *ORCA3* of *Catharanthus*^65^, contain G-box elements (CACGTG), which are necessary for JA responsiveness. Thus, HbMYC2/3/4 might interact with G-boxes in the promoter to regulate the expression of target genes. Additionally, HbMYC4 bound JRE boxes in the yeast one-hybrid assays (Fig. 4). JRE was first identified in *Catharanthus*; that JRE included a qualitative controlling element (AAACGTGCCTTT) and a quantitative controlling element (CAATAAAATATT). The bHLH transcription factor CrMYC2 could bind the JRE and activate the expression of *ORCA3*^66^. Using bioinformatics tools, we can identify which gene promoters contain G-boxes and JRE boxes, enabling us to predict *in silico* the target genes of HbMYCs.

By primarily screening the promoters of genes involved in latex biosynthesis and drainage, HbPIP2;1 was predicted as a potential target of HbMYCs due to several G-box elements existing in the core region of its promoter. As mature laticifers are devoid of plasmodesmata, the rapid exchange of water with surrounding liber cells is dependent on aquaporins embedded in the cell membrane. HbPIP2;1 is a plasma membrane-intrinsic protein. *HbPIP2;1* was up-regulated in both liber tissues and laticifers in response to bark ethrel treatment and has been proposed to play a key role in ethylene stimulation of latex yield by regulating water exchange between inner liber cells and latex cells in *H. brasiliensis*^32,55^. To determine whether *HbPIP2;1* was also the target of HbMYCs, the interaction between HbMYCs and the promoter of *HbPIP2;1* was analysed. The yeast one-hybrid assay results showed that HbMYC2/3/4 each could bind the promoter of *HbPIP2;1* (Fig. 6a). Furthermore, the dual-luciferase assay results also confirmed that HbMYC2/3/4 bound the *HbPIP2;1* promoter and up-regulated the expression of a reporter gene *in planta* (Fig. 6b and c). However, *HbPIP2;1* was negatively regulated by JA treatment and positively regulated by ET treatment (Fig. 7), which strongly suggests that there might be other ET-responsive factors that coordinate with *HbMYC2/3/4* to regulate the expression of *HbPIP2;1*. This proposal requires additional investigation.

## Methods

### Plant materials

The *H. brasiliensis* cultivar Reyan 7-33-97 used in this study was planted at the experimental farm of Hainan University. The plants were pruned each year, and epicormic shoots grew from the dormant buds on the pruned branches. RNA from the bark, latex and leaves was isolated as described to analyse the expression of genes in different tissues^67^.

### Yeast two-hybrid screening

The total RNA was isolated from the latex, bark, leaves, roots, and flowers of *H. brasiliensis*. Different RNA samples were mixed together into an RNA pool, which was used to create a “Mate & Plate™” library in accordance with the protocol of the Matchmaker™ Gold Yeast Two-Hybrid System (Clontech, USA). Additionally, HbJAZ1 was PCR-amplified by using a primer set (Supplementary Table S1-A). The PCR products were digested by *Eco*RI and *Sal*I and then ligated into pGBKT7 to generate a pGBKT7-HbJAZ1 bait vector. The yeast two-hybrid process was carried out in accordance with the Matchmaker™ Gold Yeast Two-Hybrid System (Clontech, USA).

### Transcriptional activity analysis

The positive colonies of the yeast two-hybrid screening were further cultivated on QDO/X/A media three times, after which the colonies were inoculated in QDO fluid medium for plasmid isolation. The isolated plasmids were further transformed into *E. coli* DH5a strains for sequencing. The full-length cDNAs of proteins that interacted with HbJAZ1 in the Y2H assays were obtained by *in silico* cloning procedures as previously described^31^. The coding sequences of the full-length cDNAs were cloned into a pGBKT7 vector (Clontech Inc., USA), which was further transformed into a Y2HGold yeast strain. Transcriptional activity was examined by streaking the yeast Y2HGold transformants onto SD/-Trp/-His/-Ade/X/A media (Clontech Inc., USA).

### Protoplast preparation

Protoplasts of Arabidopsis were isolated as previously described^68^. Four-week-old Arabidopsis rosette leaves were cut by a razor into 0.5–1 mm pieces and then incubated with an enzymatic hydrolysate [0.15% (w/v) pectolyase Y-23 (Yakult, Japan), 0.35% (w/v) cellulose RS (Yakult, Japan), 0.4 M mannitol, 20 mM 2-(N-morpholine)-ethanesulphonic acid (MES), 20 mM KCl and 10 mM CaCl_2_] for 2–3 hours. The protoplast was harvested by filtrating with a 45 μm syringe filter (Pall, USA) and centrifuging at 100 g at 4 °C for 8 min.

### Subcellular localization analysis

The full-length coding sequences of the *HbMYC* genes were inserted into pCAMBA1300 vectors to generate pCAMBA1300-HbMYC-GFP vectors. The reading frames of the HbMYCs and GFP were under the control of CaMV 35S promoter. The primers used are listed in Supplementary Table S1-B. The constructs and negative controls (pCAMBA1300-GFP) were transformed into Arabidopsis protoplasts as previously described^68^. The GFP fluorescence signal was visualized and imaged with a laser scanning confocal microscope (FluoView FV1000, Olympus, Japan).

### Bimolecular fluorescence complementation (BIFC)

To verify the interaction between HbJAZ1 and HbMYC3, the ORFs of HbJAZ1 and HbMYC3 were amplified by PCR and inserted into multiple clone sites (MCSs) of pSPYNE and pSPYCE to generate pSPYCE-HbJAZ1, pSPYNE-HbMYC3 and pSPYNE-HbMYC4 BIFC vectors in accordance with previously described methods^69^. The primers used are listed in Supplementary Table S1-C. Combinations of pSPYCE-HbJAZ1 and pSPYNE-HbMYC3 vectors or combinations of pSPYCE-HbJAZ1 and pSPYNE-HbMYC4 vectors were co-transformed into Arabidopsis protoplasts in accordance with previously described methods^68^. YFP fluorescence was visualized and imaged by a laser scanning confocal microscope (FluoView FV1000, Olympus, Japan).

### Quantitative real-time PCR (qRT-PCR)

All RNA samples were treated with RQ1 RNase-free DNase I (Promega) to remove DNA contamination, and the quality and concentration of the DNaseI-treated total RNA were both checked by agarose gel electrophoresis and measured by spectrophotometry. Two micrograms of DNase I-treated total RNA was used as template for first-strand cDNA synthesis in accordance with the manufacturer’s instructions (RevertAid™ First Stand cDNA Synthesis Kit, Fermentas, LT-2028 Vilnius, Lithuania). The qRT-PCR assays were performed using an ABI-7500 Real-Time PCR apparatus with SYBR Green I dye (Takara). The cDNA encoding 18 S rRNA was chosen as a reference gene using GeNorm software. The efficiency of each primer pair was evaluated before PCR. The primers used are listed in Supplementary Table S1-E. PCR was performed as follows: 3 min at 95 °C, followed by 40 cycles of denaturation at 95 °C for 15 s, annealing at 58 °C for 15 s, and extension at 72 °C for 20 s. The relative abundance of transcripts was automatically calculated using the 2^−△△CT^ method by the ABI-7500 software using the 18S rRNA gene as an internal standard. All experiments were performed with three independent biological replicates and three technical repetitions. SE calculations and ANOVA were used for statistical and significance analyses, respectively.

### Yeast two-hybrid assays

The cloned full-length CDSs of the HbJAZ genes were PCR-amplified and inserted into a pGBKT7 vector to generate a bait vector. The bait vector was first transformed into a yeast Y2H gold strain to test for toxicity and autotranscription activity as described by the manufacturer (Cat. No. 630489,Clontech, Inc., USA). Subsequently, the CDSs of the HbMYC2/3/4 genes were further fused into a pGADT7 vector to generate prey plasmid. The primers used are listed in Supplementary Table S1-A. The bait vector and prey vector were subsequently transformed into Y187 and Y2Hgold strains, respectively. The yeast two-hybrid process was performed by mating together the Y187 and Y2Hgold strains, after which they were plated onto QDO/X/A (SD-Trp/-Leu/-Ade/-His/X/A) selection media.

### Yeast one-hybrid assays

The HbMYC2/3/4-interacting cis-elements were screened using a Matchmaker Gold Y1H Screening system (Clontech). Forward and reverse nucleotide oligos for each cis-element, e.g., JREs (JA-responsive elements), GCC boxes, ABREs (ABA-responsive elements), ERE (ethylene-responsive elements), G-boxes, CACG boxes, and DREs (dehydration-responsive elements), were synthesized (listed in Supplementary Table S1-D). Each pair of oligo sequence was annealed and ligated into a pAbAi vector. The resulting pAbAi-bait plasmid was transformed into a Y1HGold strain to generate a bait reporter strain. The full-length CDSs of the HbMYC2/3/4 genes were then amplified with gene-specific primers (Supplementary Table S1-A). The PCR products were subsequently cloned into the pGADT7 prey vectors (Clontech), and the resulting prey vectors were transferred into the aforementioned bait reporter strains. The transformed cells were then grown on SD/–Leu plates at 30 °C for 3 days, after which time the cells were collected. The resuspended cells were then plated onto SD/–Leu media containing different AbA concentrations.

### Dual-luciferase assays

The promoter sequence of *HbPIP2;1* was amplified by PCR using the genomic DNA of cultivar Reyan 7-33-97 and was inserted into the MCSs of pSP-luc+NF plasmids. The full-length CDSs of *HbMYC2/3/4* were amplified and inserted into PBI121 vectors. The specific primers used are listed in Supplementary Table S1-F. Two types of plasmids were transiently co-transferred into protoplasts by the polyethylene glycol (PEG)-mediated method. Sixteen hours after incubation in the dark at 20 °C, the protoplasts were harvested. The luciferase activities were subsequently quantified using a dual-luciferase assay kit (Promega, USA) and detected by using a GloMax® 96 microplate luminometer (Promega, USA).

## Electronic supplementary material

Dataset1
