# Supplementary material for: Identification of JAZ-interacting MYC transcription factors involved in latex drainage in Hevea brasiliensis
Source: Sci Rep. 2018 Jan 17;8:909. doi: 10.1038/s41598-018-19206-3 (PMC5772448; doi:10.1038/s41598-018-19206-3)
Supplement: Supplementary file 1 — Dataset1 [file 41598_2018_19206_MOESM1_ESM.doc]

Identification of JAZ interacted MYC transcription factors involved in latex drainage in *Hevea brasiliensis*

Jinling Zhai^§^, Hui Hao^§^, Hua Xiao, Yuxin Cao, Xiangui Lin, Xi Huang^*^

Hainan Key Laboratory for Sustainable Utilization of Tropical Bioresources, Institute of Tropical Agriculture and Forestry, Hainan University, Haikou 570228, P. R. China

# § These authors contributed equally to this work.

^*^ Corresponding author:

Mailing address: College of Tropical Agriculture and Forestry, Hainan University, Renmin RD 58, 570228 Haikou, P. R. China.

Fax: 0086-898-66258650

Tel: 0086-898-66279271

E-mail: [xihuang@hainu.edu.cn](mailto:xihuang@hainu.edu.cn)

**Table S1. Primers used in this work.**

| **Primer name** | **Sequences (5`—3`)** |
| --- | --- |
| **a. Primers for constructing Yeast-two-hybrid vectors.** | |
| HbMYC2 for | ggGGATCCgtatgacggactatcggataacg |
| HbMYC2 rev | acCTGCAGtcaggtgtcaccaactttggt |
| HbMYC3 for | ttcATATGaagattgagataggtatg |
| HbMYC3 rev | ttGGATCCctatccgacggatgacaattg |
| HbMYC4 for | ggGGATCCgtatgggtgagaaattttgggt |
| HbMYC4 rev | tcGTCGACtcatttagaaagggcagtct |
| HbJAZ1 for | aatGAATTCatggctggctcgccggaatttg |
| HbJAZ1 rev | aaGTCGACctgcaaagattgaccagccaggc |
| HbJAZ2 for | ttCATATGgcgaacttggtccagaat |
| HbJAZ2 rev | ttGGATCCctataatttaagttcaagct |
| HbJAZ3 for | aaGAATTCatggctggttcgcccgatttcgt |
| HbJAZ3 rev | aaGGATCCtcaaatatgattagcaatcgtgt |
| HbJAZ5 for | aaGAATTCatggcaaacttggttcacaaatc |
| HbJAZ5 rev | aaGGATCCctataacttaagctcaagctgt |
| HbJAZ6 for | tacgctCATATGaggaggaactgcaatcttgaactccg |
| HbJAZ6 rev | aaGGATCCtcaatggtcgaccctgcatgca |
| HbJAZ7 for | ttCATATGtcgaggaactgcaatctt |
| HbJAZ7 rev | ttGAATTCtcaatggtgatcgaccctgca |
| HbJAZ8 for | ttCATATGagacgaaactgcaatct |
| HbJAZ8 rev | ttGGATCCttaatgattgtaaggagaaatc |
| HbJAZ9 for | ttCATATGgagagagattttatgggt |
| HbJAZ9 rev | ttGGATCCtcattctattggattctgca |
| HbJAZ10 for | ttCATATGtcgagagcaactgtcga |
| HbJAZ10 rev | ttGGATCCctataaacgacaaacagatttg |
| HbJAZ11 for | ttCATATGgagggtgattctgattc |
| HbJAZ11 rev | ttGGATCCttaagcaaggttggcagtaac |
| HbJAZ12 for | tacgctCATATGgagggtgaatctgattcgtat |
| HbJAZ12 rev | aaGAATTCttaagcaaggttggcagtaacct |
|  |  |
| **b. Primers for construction of subcellular localization.** | |
| HbMYC2 for | gtACTAGTatgacggactatcggata |
| HbMYC2 rev  HbMYC3 for  HbMYC3 rev | ttGGATCCggtgtcaccaactttggt  gtACTAGTatgaagattgagataggtatg  ttGGATCCtccgacggatgacaattg |
| HbMYC4 for | gtACTAGTatgggtgagaaattttgggt |
| HbMYC4 rev | ttGGATCCtttagaaagggcagtctcca |
|  |  |
| **c. Primers for BIFC assay.** | |
| HbMYC3 for | ttcGGATCCatgaagattgagataggtatg |
| HbMYC3 rev  HbMYC4 for  HbMYC4 for | ttCTCGAGctatccgacggatgacaattg  ggGGATCCatgggtgagaaattttgggt  ttCTCGAGtcatttagaaagggcagtct |
| HbJAZ1 for | aatGGATCCatggctggctcgccggaatttg |
| HbJAZ1 rev | aaCTCGAGctgcaaagattgaccagccaggc |
|  |  |
| **d. The sequences of cis-elements used for constructing pAbAi bait vectors.** | |
| JRE for | ATTTTTCAATAAAATATTTATCCATTTTTCAAACGTGCCTTT |
| JRE rev | TCGAAAAGGCACGTTTGAAAAATGGATAAATATTTTATTGAAAAATGTAC |
| GCC box for | ATTTTTGCCGCCTATCCATTTTTCGCCGCC |
| GCC box rev | TCGAGGCGGCGAAAAATGGATAGGCGGCAAAAATGTAC |
| ABRE for | ATTTTTACGTGTCTATCCATTTTTCACGTGGC |
| ABRE rev | TCGAGCCACGTGAAAAATGGATAGACACGTAAAAATGTAC |
| ERE for | ATTTTTATTTCAAATATCCATTTTTCAAATTCAAA |
| ERE rev | TCGATTTGAATTTGAAAAATGGATATTTGAAATAAAAATGTAC |
| G-box for | ATTTTTCACGTGTATCCATTTTTCCACGTG |
| G-box rev | TCGACACGTGGAAAAATGGATACACGTGAAAAATGTAC |
| CACG for | ATTTTTCACGTATCCATTCACGCATTTTCACG |
| CACG rev | TCGACGTGAAAATGCGTGAATGGATACGTGAAAAATGTAC |
| DRE for | ATTTTTCCGACTATCCATTTTTCCCGAC |
| DRE rev | TCGAGTCGGGAAAAATGGATAGTCGGAAAAATGTA |
|  |  |
| **e. Primers for qRT-PCR** | |
| 18S-rRNA for | GTGGAAGAGGTTCAGAAGAG |
| 18S-rRNA rev | TTCAGCCTTGCGACCATAC |
| HbMYC2 for | GGTGGGTTCATGGCCGA |
| HbMYC2 rev | GCCATTTGTAGTGCCA |
| HbMYC3 for | ATTTTGATGGTTCAGTCA |
| HbMYC3 rev | CACATTGGAAAACAGGGA |
| HbMYC4 for | GCAAAAACTATATTTG |
| HbMYC4 rev | TATCTTCAACCTTAGGAGA |
|  |  |
| **f. Primer for Dual-luciferase assay** | |
| HbPIP2-P for | gaaGATCTAaaaaattatatttta |
| HbPIP2-P rev | cccAAGCTTtaggtaccgtgacagg |
| HbMYC2 for | cgGGATCCatgacggactatcggataacgccgac |
| HbMYC2 rev | tcccCCCGGGtcaggtgtcaccaactttggttgat |
| HbMYC3 for | gcTCTAGAatgaagattgagataggtatgggagg |
| HbMYC3 rev | cgGGATCCctatccgacggatgacaattgctgt |
| HbMYC4 for | gcTCTAGAatgggtgagaaattttgggtgaatga |
| HbMYC4 rev | cgGGATCCtcatttagaaagggcagtctccagt |
